# Release of gp120 Restraints Leads to an Entry-Competent Intermediate State of the HIV-1 Envelope Glycoproteins

**DOI:** 10.1128/mBio.01598-16

**Published:** 2016-10-25

**Authors:** Alon Herschhorn, Xiaochu Ma, Christopher Gu, John D. Ventura, Luis Castillo-Menendez, Bruno Melillo, Daniel S. Terry, Amos B. Smith, Scott C. Blanchard, James B. Munro, Walther Mothes, Andrés Finzi, Joseph Sodroski

**Affiliations:** a Department of Immunology Cancer and Virology, Dana-Farber Cancer Institute, Boston, Massachusetts, USA; b Department of Microbiology and Immunobiology, Harvard Medical School, Boston, Massachusetts, USA; c Department of Microbial Pathogenesis, Yale University School of Medicine, New Haven, Connecticut, USA; d Department of Chemistry, University of Pennsylvania, Philadelphia, Pennsylvania, USA; e Department of Physiology and Biophysics, Weill Cornell Medical College of Cornell University, New York, New York, USA; f Department of Molecular Biology and Microbiology, Tufts University School of Medicine and Sackler School of Graduate Biomedical Sciences, Boston, Massachusetts, USA; g Centre de Recherche du CHUM and Department of Microbiology, Infectiology and Immunology Université de Montréal, Montreal, Quebec, Canada; Department of Microbiology and Immunology, McGill University, Montreal, Quebec, Canada; h Department of Immunology and Infectious Diseases, Harvard T. H. Chan School of Public Health, Boston, Massachusetts, USA

## Abstract

Primary human immunodeficiency virus (HIV-1) envelope glycoprotein (Env) trimers [(gp120/gp41)_3_] typically exist in a metastable closed conformation (state 1). Binding the CD4 receptor triggers Env to undergo extensive conformational changes to mediate virus entry. We identified specific gp120 residues that restrain Env in state 1. Alteration of these restraining residues destabilized state 1, allowing Env to populate a functional conformation (state 2) intermediate between state 1 and the full CD4-bound state (state 3). Increased state 2 occupancy was associated with lower energy barriers between the states. State 2 was an obligate intermediate for all transitions between state 1 and state 3. State 2-enriched Envs required lower CD4 concentrations to trigger virus entry and more efficiently infected cells expressing low levels of CD4. These Envs were resistant to several broadly neutralizing antibodies and small-molecule inhibitors. Thus, state 2 is an Env conformation on the virus entry pathway; sampling state 2 increases the adaptability of HIV-1 to different host cell receptor levels and immune environments. Our results provide new insights into the conformational regulation of HIV-1 entry.

## IMPORTANCE

The envelope glycoproteins (Env) of HIV-1 mediate virus entry and are the sole targets of neutralizing antibodies. Understanding the way that Env promotes HIV-1 entry can expedite drug and vaccine development. By destabilizing Env, we found that it assumes an intermediate state that is functional and obligate for transitions to entry-competent conformations. Increased sampling of this state enhances the ability of HIV-1 to infect cells that express low levels of the CD4 receptor and allows the virus to evade neutralizing antibodies and small-molecule inhibitors. These findings provide new mechanistic insights into the function and inhibition of HIV-1 Env and will contribute to ongoing therapeutic and prevention efforts to combat HIV-1.

## INTRODUCTION

The entry of human immunodeficiency virus (HIV-1) into host cells is mediated by the viral envelope glycoprotein (Env) trimer. HIV-1 Env is composed of three gp120 exterior subunits noncovalently associated with three gp41 transmembrane subunits (1–3). Binding of gp120 to the CD4 receptor triggers the transition of Env from a metastable, high-potential energy state to downstream conformations. CD4-induced (CD4i) gp120 transitions include a repositioning of the V1/V2 and V3 loops and formation of the bridging sheet and coreceptor binding site (4–12). The heptad repeat (HR1) coiled coil in the gp41 ectodomain is also formed and exposed after CD4 binding (13–16). Subsequent binding to the CCR5 or CXCR4 coreceptor promotes the formation of a stable gp41 six-helix bundle, composed of the HR1 and HR2 heptad repeats, that mediates the fusion of the viral and target cell membranes (17–21).

The mature, unliganded Env of most primary clinical HIV-1 isolates assumes a “closed” conformation of the gp120 subunits at the trimer apex (22–27). Here, we refer to the native “closed” Env conformation as state 1. CD4 binding rearranges the gp120 V1/V2 and V3 loops at the trimer apex, thus “opening” the HIV-1 Env trimer to form the prehairpin intermediate (22), referred to here as state 3. Env transitions between state 1 and state 3 must be tightly regulated to allow entry into cells with different levels of receptor, while sequestering conserved Env elements from host neutralizing antibodies (NAbs). HIV-1 strains differ in the propensity of their Envs to make these transitions and sample downstream conformations; this property contributes to different requirements for target cell CD4 levels and different sensitivities to host neutralizing antibodies, small-molecule entry inhibitors, and incubation in the cold (28–37). Information on the entry-related transitions of HIV-1 Env can be obtained by identifying and studying functional conformational intermediates.

Here, starting with the difficult-to-neutralize primary HIV-1_JR-FL_, we identify key residues in the gp120 V1/V2 elements that restrain Env movement from state 1 and thus regulate Env transitions to downstream conformations. Alteration of these restraining residues resulted in extensive conformational changes, generating entry-competent Envs that sample an intermediate conformation (state 2) between state 1 and the full CD4-bound state (state 3). We determined the relative free energies of these conformations and demonstrated that changes in the restraining residues lowered the activation barriers separating these states. Env variants in state 1 and state 2 exhibited distinct patterns of susceptibility to inhibition by ligands that preferentially recognize particular Env conformations. Lastly, modulating the continuous range of transitions between Env states allowed us to evaluate the conformational preferences of broadly neutralizing antibodies (bNAbs); we then used selective bNAbs to study the functional conservation of a restraining gp120 V2 residue among HIV-1 subtypes.

## RESULTS

### Entry-competent intermediate states of HIV-1 Env.

**FIG 1:**
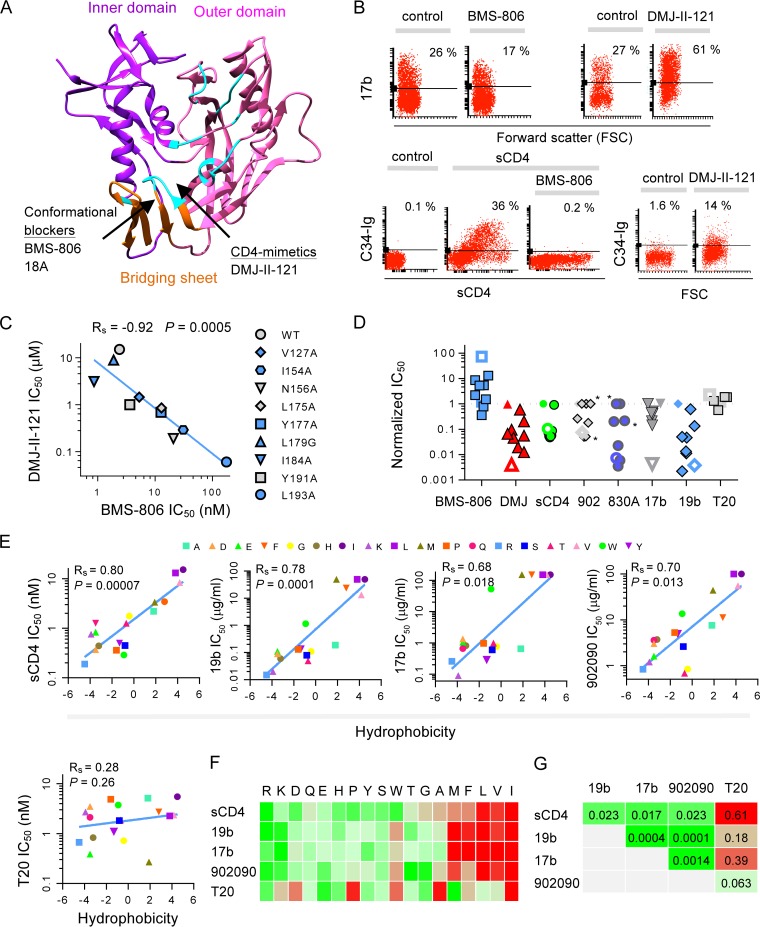
Specific amino acid residues in the gp120 V1/V2 region regulate HIV-1 Env conformational transitions. (A) The binding sites of the small-molecule HIV-1 entry inhibitors used in this study are indicated by arrows on a ribbon structure of the gp120 core (PDB entry 1RZK). The gp120 core inner domain (purple), outer domain (magenta), and bridging sheet (orange) are shown. The location of the inhibitor binding sites is based on available crystal structures (38, 39). The gp120 residues contacting CD4 (12) are colored in cyan. (B) Flow cytometric analysis of the effect of the CD4-mimetic compound (CD4MC) DMJ-II-121 (100 μM) and the BMS-806 blocker of conformational change (1 μM) on the conformation of the HIV-1_JR-FLΔCT_ Env expressed on the cell surface. Structural rearrangements were measured using the CD4i 17b antibody and C34-Ig, which binds the gp41 HR1 region. (C) Inverse relationship between the sensitivity of HIV-1_JR-FL_ V1/V2 Env mutants to inhibition by a CD4MC (DMJ-II-121) and the sensitivity to a blocker of conformational change, BMS-806. Spearman’s Rho coefficient and two-tailed *P* value are reported. (D) Normalized IC_50_s (IC_50_ mutant/IC_50_ WT) of inhibition by BMS-806 and DMJ-II-121 (from panel C), sCD4, antibodies 902090, 830A, 17b, and 19b, and the T20 peptide. The WT Env is depicted by a filled symbol with no border, the L193A variant is depicted by an open symbol, and Env variants with alterations in the putative binding site of the antibodies are marked with an asterisk. 902 = the 902090 antibody; DMJ = DMJ-II-121. (E) Relationship between the hydrophobicity of gp120 residue 193 and HIV-1 inhibition by conformation-sensitive Env ligands. The hydrophobicity of substituted amino acids was assigned based on the work of Kyte and Doolittle (42). Spearman’s Rho coefficient and two-tailed *P* values are reported for each analysis. (F) A heat map of the inhibition (on a logarithmic scale) of Env variants with the specified changes in residue L193 by the indicated Env ligands. Amino acid substitutions, ranked according to hydrophobicity, are shown on the top of the heat map in single-letter code. Increased-sensitivity data are indicated in green. (G) Correlations among the susceptibilities of the HIV-1 L193 mutant virus panel to inhibition by different conformation-sensitive Env ligands were evaluated. Two-tailed *P* values are reported for each correlation, with green coloring indicating statistical significance (*P* < 0.05) and red coloring a lack of statistical significance. sCD4, soluble CD4. Results shown are representative (B) or averages (C to F) of those obtained in two or three independent experiments.

We identified two groups of HIV-1 entry inhibitors and used them as chemical probes to study Env conformation: (i) CD4-mimetic compounds (CD4MCs) such as DMJ-II-121 and (ii) blockers of conformational change such as BMS-806 and 18A (16, 37, 38). CD4MCs and blockers of conformational change bind to different sites on HIV-1 gp120 (Fig. 1A) (38, 39). We found that these two groups of compounds exert opposing effects on structural rearrangements of Env (Fig. 1B). The CD4MC DMJ-II-121 induced the formation/exposure of the 17b epitope (near the coreceptor binding site of gp120 [40]) and the gp41 HR1 (recognized by the C34-Ig protein [16]). In contrast, the blocker of conformational change BMS-806 decreased 17b binding and blocked soluble CD4 (sCD4)-induced formation/exposure of gp41 HR1 (16). Thus, CD4MCs such as DMJ-II-121 induce an Env conformation similar to that induced by CD4 and therefore exhibit greater potency against Envs in the CD4-bound conformation (state 3). In contrast, blockers of conformational change such as BMS-806 and 18A demonstrate increased potency against Envs in state 1 (37).

We next used our chemical probes to identify specific amino acid changes that alter the conformation of the functional Env trimer on virions. On the basis of the observation that the HIV-1 gp120 V1/V2 and V3 regions contribute to contacts among the gp120 protomers of the Env trimer (24–27), we hypothesized that some V1/V2 changes might compromise the maintenance of state 1 and induce transitions to downstream conformations. By studying a large panel of HIV-1_JR-FL_ V1/V2 mutants, we identified a subset of mutants that exhibited increased virus sensitivity to soluble CD4 (sCD4) and to incubation in the cold; these two properties were previously shown to be associated with Envs that are more prone to undergo conformational change (41) (see Table S1 in the supplemental material). Measuring virus sensitivity to our chemical probes revealed an inverse relationship between the sensitivities of the V1/V2 mutants to the CD4MC DMJ-II-121 and the blocker of conformational change BMS-806 (Fig. 1C). As the V1/V2 element is not directly involved in binding these inhibitors (38, 39), the observed pattern of altered sensitivity likely results from changes in Env conformation. The observed increase in resistance to BMS-806, which prefers state 1, along with hypersensitivity to the CD4MC DMJ-II-121, which preferentially recognizes the CD4-bound state, suggested a transition of these mutants to downstream conformations. Consistent with such conformational changes, the V1/V2 mutants exhibited increased susceptibility to Env ligands recognizing downstream conformations (Fig. 1D; see also Table S1). These ligands included sCD4 and the following monoclonal antibodies (Mabs): 19b, directed against the gp120 V3 loop; MAb 902090 and Fab 830A, directed against a V2 β-barrel; and 17b, a CD4-induced (CD4i) MAb (see Table S2). The V1/V2 mutants were not generally more sensitive to the T20 peptide (Fig. 1D); therefore, unlike Envs in the full CD4-bound state (32), the mutants do not spontaneously expose the gp41 HR1 coiled coil. Thus, these mutants apparently sample conformations that are distinct from state 1 and yet differ from the full CD4-bound state (state 3).

### Role of leucine 193 in maintaining state 1.

Of the V1/V2 mutants, L193A exhibited the greatest phenotypic differences from wild-type (WT) Env with respect to virus inhibition by conformation-sensitive Env ligands (Fig. 1C and D). The L193A mutant was 75-fold more resistant to the blocker of conformational change BMS-806 than WT Env and was approximately 250-fold more sensitive to the CD4MC DMJ-II-121. The L193A virus was hypersensitive to neutralization by the 902090 and 830A anti-V2 antibodies, the 19b anti-V3 antibody, and the 17b CD4i antibody. Notably, the L193A change induced these specific conformational effects while maintaining productive infectivity as well as efficient Env processing and syncytium-forming capacity (see Table S1 in the supplemental material). The simultaneous and substantial exposure of multiple elements in the L193A Env suggested that Leu 193 may represent an amino acid residue that maintains Env in state 1. To test this hypothesis, multiple amino acid changes were introduced into Env residue 193 and their effect was measured. We observed an inverse correlation between the hydrophobic character of residue 193 (estimated according to Kyte and Doolittle [42]) and susceptibility of the corresponding mutant viruses to inhibition by ligands that recognize downstream Env conformations (Fig. 1E; see also Fig. S2 and S3). Nonhydrophobic substitutions resulted in up to 100-fold increases in virus sensitivity to sCD4 and a V2 MAb and in more than 1,000-fold increases in sensitivity to a V3 and CD4i MAb; sensitivity to the T20 peptide was not significantly different from that of the WT virus (Fig. 1E to G). Thus, when residue 193 is not hydrophobic, the maintenance of state 1 is compromised, allowing Env to sample an intermediate conformation that is distinct from, but shares some antigenic features with, the CD4-bound conformation.

**FIG 2:**
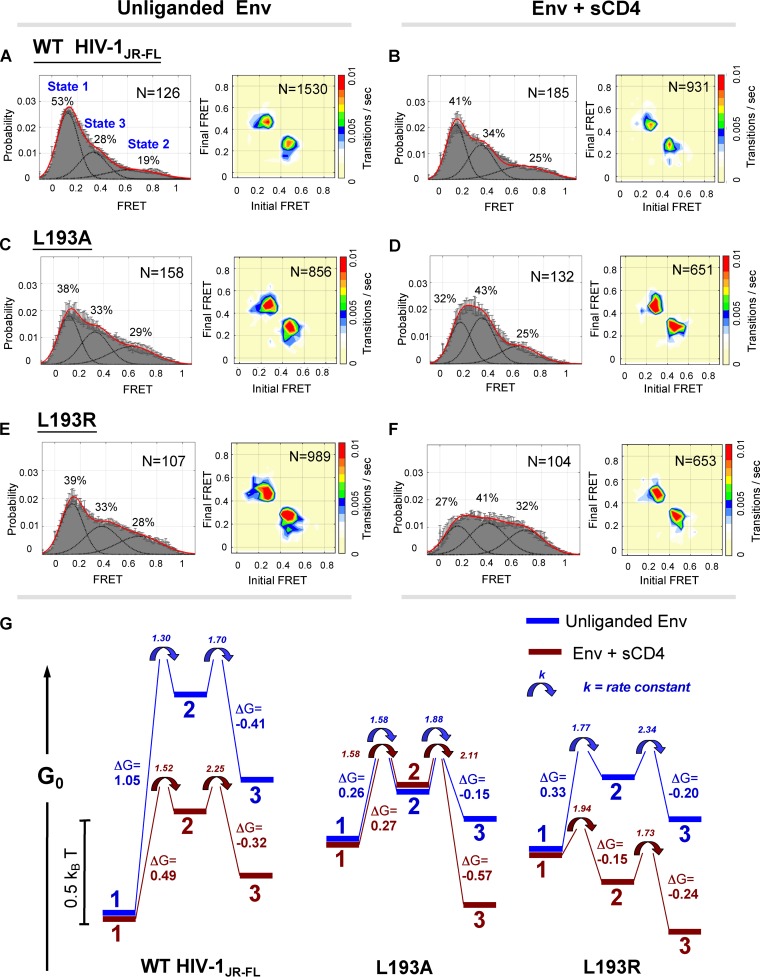
Single-molecule FRET analysis of the WT and leucine 193 HIV-1 Env variants. (A to F) Single-molecule fluorescence resonance energy transfer (smFRET) probes were placed in the gp120 V1 and V4 loops of WT or L193 mutant HIV-1_JR-FL_ Envs. FRET trajectories were compiled into population FRET histograms and fitted to the Gaussian distributions associated with each conformational state, according to a hidden Markov model (43). The percentages of the population that occupied each state and the numbers of molecules analyzed are shown and represent averages of results of two independent experiments. Transition density plots (TDPs) are shown on the right. Note that the densities for the transitions between state 1 and state 2 and between state 2 and state 3 were readily detectable in the TDPs, whereas the densities for the transitions between state 1 and state 3 were not evident. This suggests that transitions between state 1 and state 3 occur through state 2. The results for the unliganded Envs (A, C, and E) and after incubation of the Envs with D1D2 sCD4 (B, D, and F) are shown. (G) The relative free energies of states 1, 2, and 3 for the unliganded (blue) and sCD4-bound (red) Envs. The placement of the energy diagrams for each Env variant on the absolute G_0_ scale is arbitrary. Note the similarity in the energy landscape of the unliganded L193 Env and the sCD4-bound WT Env proteins. The forward rate constants (1/s) are indicated adjacent to the arrows at each transition point.

We next used single-molecule fluorescence resonance energy transfer (smFRET) to investigate the conformational dynamics of the WT, L193A, and L193R HIV-1_JR-FL_ viruses. smFRET analysis previously demonstrated that HIV-1 WT Env can sample at least three conformations detectable with FRET probes in the V1 and V4 variable regions of gp120 (43). Two conformations were defined with high confidence: state 1 (low FRET) represents the unliganded Env conformation, and state 3 (intermediate FRET) represents the full CD4-bound conformation. The identity and functional significance of the high-FRET state are currently unknown. This state was hypothesized to represent either a CD4-bound conformation or a previously uncharacterized and necessary structural intermediate (43). We defined state 2 as this high-FRET conformation and used the leucine 193 mutants to investigate its nature. The WT HIV-1_JR-FL_ Env primarily occupied the “closed” conformation (state 1) and made infrequent transitions from this state (Fig. 2A). Incubation with sCD4 moderately shifted some of the WT HIV-1_JR-FL_ Env to downstream conformations (Fig. 2B). Notably, transitions of WT Env from state 1 to state 3 occurred exclusively through state 2, suggesting the potential functional importance of Env transitions through state 2. Compared with the WT Env, the unliganded L193 mutants exhibited a substantial shift in the occupancy of the conformational states. The occupancy of state 1 was lowered and the occupancy of state 2 was increased (with a smaller increase for state 3) for the unliganded L193 Envs relative to the WT Env (Fig. 2A, C, and E). Notably, the distribution of conformations of both L193 mutants in their unliganded state was similar to that of the WT Env incubated with sCD4. Transitions among the different conformations of the unliganded L193A and L193R Envs were more frequent than those detected for the WT Env, which was reflected in the higher rate constants calculated for transitions between conformational states of the L193 mutants (Fig. 2A, C, and E; see also Fig. S4 and Table S3 in the supplemental material). The higher rate constants indicate that the activation barriers separating the conformational states of the L193 mutants are lower than those of the WT Env. The magnitude of the increase in transitions of the L193A and L193R mutants was in agreement with their relative sensitivities to ligands recognizing downstream Env conformations. The addition of sCD4 to the L193 mutants resulted in profound transitions into downstream states, shifts that were significantly greater than that observed for the WT Env incubated with sCD4 (Fig. 2B, D, and F). CD4 binding to the L193A Env increased the occupancy of state 3; CD4 binding to the L193R Env increased the occupancies of both state 2 and state 3. As was observed for the WT Env, transitions of the L193A and L193R Envs from state 1 to state 3 occurred exclusively through state 2. Thus, alteration of the hydrophobic Leu 193 decreases the energy barriers between state 1 and downstream states, increasing the propensity of the mutant Env to sample state 2 and state 3, both spontaneously and after the binding of CD4 (Fig. 2G). These results relate functional Env phenotypes to conformational states defined by smFRET and illustrate the relationship between viral phenotypes and thermodynamic parameters. Because the Env L193 mutants retain the ability to bind receptors and mediate membrane fusion, our data support a model in which state 2 is a functional intermediate on the virus entry pathway.

### Enrichment of state 2 lowers the CD4 concentration required for HIV-1 entry.

**FIG 3:**
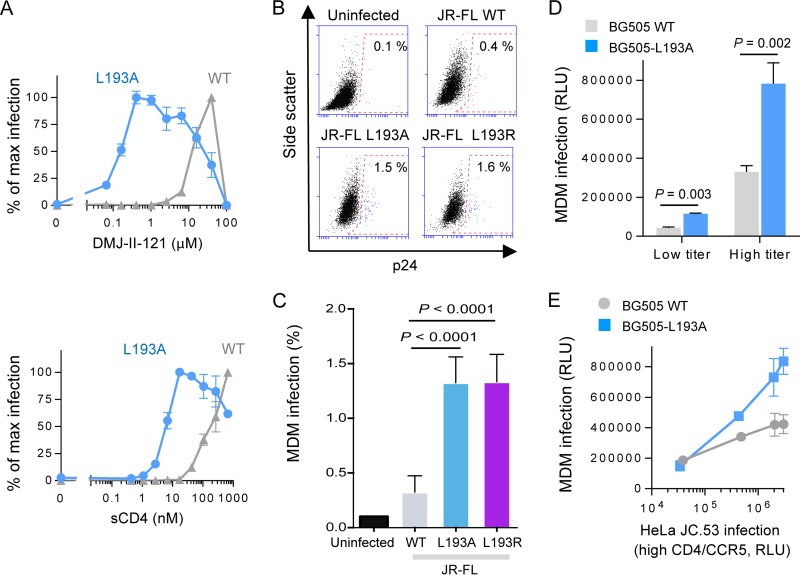
Effect of changes in Env on CD4 dependence and infectivity of monocyte-derived macrophages (MDMs). (A) Recombinant viruses carrying the specified HIV-1_JR-FL_ Env were incubated with CD4-negative, CCR5-expressing Cf2Th cells in the presence of CD4MC DMJ-II-121 or sCD4 at the indicated concentrations. The percentage of maximal infection for each virus variant is reported. Maximal (max) infection levels after DMJ-II-121 addition were 728,752 and 237,686 relative luciferase units (RLU) for viruses with the WT and L193A Envs, respectively. (B and C) Infection of MDMs by WT HIV-1_JR-FL_ and L193 mutant viruses was measured as described in Materials and Methods. (D) Infection of MDMs by HIV-1_BG505_ and the HIV-1_BG505_ L193A viruses. (E) Infection of MDMs versus HeLa JC.53 cells, which express high levels of CD4 and CCR5 (61), by HIV-1_BG505_ and the HIV-1_BG505_ L193A viruses. Results shown are averages (A and C) or representative (B, D, and E) of those obtained in 2 to 5 independent experiments.

Changes in Leu 193 in the V1/V2 loop of gp120 resulted in the highest increases in the sensitivities of the resulting virus variants to ligands recognizing downstream conformations. The inferred increase in Env sampling of downstream states is expected to enhance the ability of these HIV-1 variants to infect cells with lower levels of CD4 (28, 29, 31, 32). To evaluate the requirement of CD4 for infection, we incubated viruses displaying the WT and L193A Envs with CD4-negative, CCR5-expressing cells and measured the ability of different concentrations of the CD4MC DMJ-II-121 or sCD4 to activate infection (Fig. 3A). Neither virus efficiently infected the cells in the absence of a CD4 mimetic (data not shown). The L193A mutant required significantly less CD4MC or sCD4 to trigger virus entry than the WT Env, confirming its enhanced propensity to transition into the CD4-bound conformation.

As the ability to enter cells with low CD4 expression is one requirement for macrophage tropism (34, 44–47), we asked if the L193 mutants exhibited an increased ability to infect primary human macrophages compared with the wild-type virus. We generated wild-type and L193A and L193R mutant HIV-1_JR-FL_, normalized the virus titers according to infectivity on TZMbl cells, and infected human monocyte-derived macrophages. The ability of the two state-2-enriched mutants to infect primary macrophages was increased, on average, ~4-fold over that of the wild-type HIV-1_JR-FL_ (Fig. 3B and C). A similar phenotype was observed for a recombinant HIV-1 with the L193A Env variant of HIV-1_BG505_, a non-macrophage-tropic strain (Fig. 3D and E). These data relate shifts in the energy landscapes of HIV-1 Env variants toward state 2 and state 3 to an increased ability of the virus to infect cells with low levels of CD4. Additional requirements shaped by the cellular and immunological environment ultimately determine HIV-1 macrophage tropism *in vivo*.

### Conformational preferences of broadly neutralizing antibodies.

**FIG 4:**
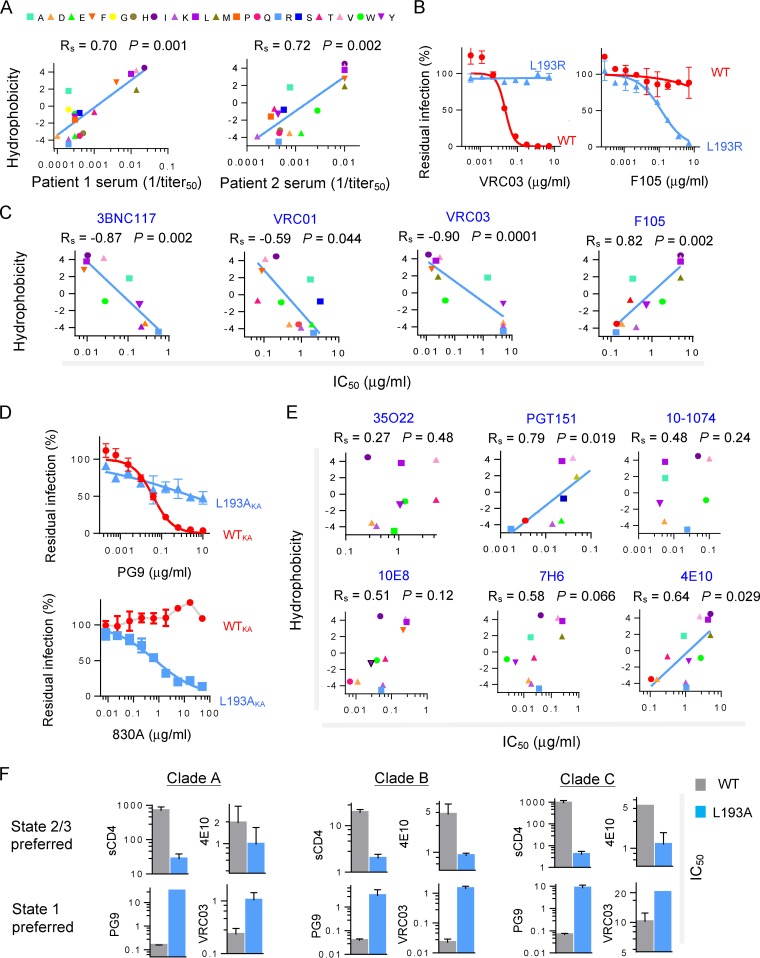
Neutralization of HIV-1 variants differing in conformational state by human antibodies elicited during infection. (A) Recombinant viruses with Envs containing substitutions in residue 193 were tested for sensitivity to neutralization by antibodies. The relationship between the hydrophobicity of residue 193 and sensitivity to neutralization by polyclonal sera (PS) from two HIV-1-infected individuals is shown. (B) Neutralization of WT and L193R HIV-1_JR-FL_ by two different types of CD4-BS antibodies: the bNAb VRC03 and the weakly neutralizing F105 antibody. (C) Relationship between the hydrophobicity of Env residue 193 and HIV-1_JR-FL_ sensitivity to neutralization by four CD4-BS antibodies: three bNAbs (3BNC117, VRC01, and VRC03) and one weakly neutralizing antibody, F105. (D) Sensitivity of viruses with the indicated Env variants to neutralization by the PG9 antibody. The Env variants tested have changes distant from the defined V1/V2 binding site of the PG9 antibody (62). All these variants have, in addition, the E168K-plus-N188A changes that are required for the binding of PG9 to the HIV-1_JR-FL_ Env (51). (E) The relationship between the hydrophobicity of Env residue 193 and HIV-1_JR-FL_ sensitivity to neutralization by bNAbs directed against gp120-gp41 hybrid epitopes (35O22 and PGT151), a V3 glycan-dependent epitope (10-1074), and gp41 MPER epitopes (10E8, 7H6, and 4E10) is shown. (A, C, and E) Spearman’s Rho coefficient and two-tailed *P* values are shown. (F) Conformation-selective bNAbs and sCD4 were used to test the sensitivity of the WT and the L193A variant of HIV-1 strains BG505 (clade A), JR-FL (clade B), and ZM53M.PB12 (clade C). For PG9 neutralization, the E168K + N188A mutant of HIV-1_JR-FL_ was used. Reported IC_50_ units are nanomolar (nM) for sCD4 and micrograms per milliliter (μg/ml) for the bNAbs. Data shown are averages of results obtained in two or three independent experiments.

The ability to modulate the energy landscape of the HIV-1_JR-FL_ Env allowed us to evaluate the conformational preferences of antibodies generated in humans during HIV-1 infection. We examined the ability of polyclonal sera from two HIV-1-infected individuals to neutralize the panel of Leu 193 mutants. In both cases, the neutralization sensitivity of the mutants inversely correlated with the hydrophobicity of Leu 193 (Fig. 4A) (42). The sensitivity of the virus panel to the polyclonal sera correlated with the sensitivity to sCD4, 19b, 17b, and 902090 but not with the sensitivity to T20 (see Fig. S2 in the supplemental material). Thus, most of the neutralizing antibodies in these sera are directed against Env epitopes that are better exposed in state 2 than in state 1.

Broadly neutralizing antibodies (bNAbs) are elicited in only a minority of HIV-1-infected humans and after a long period of infection (48). We used the HIV-1_JR-FL_ Leu 193 mutant panel to evaluate bNAb selectivity for specific Env conformations. Three different patterns of conformational selectivity among these antibodies were identified. The first group included the CD4 binding site (CD4-BS) and V2 quaternary bNAbs and showed a strict preference for state 1. The WT Env was efficiently inhibited by these bNAbs; however, nonhydrophobic substitutions at residue 193 generated Env variants that were significantly more resistant to neutralization by these bNAbs (Fig. 4B to D; see also Fig. S5 in the supplemental material). In contrast, weakly neutralizing antibodies directed against related sites, namely, F105 (against the CD4-BS) and 830A (against the V2 β-barrel [49]), showed the opposite preference, inhibiting only viruses with Envs that moved from state 1 to downstream conformations. The preference of the CD4-BS and V2 quaternary bNAbs for state 1 is consistent with the occlusion or disruption of their respective epitopes by CD4 binding (37, 50, 51).

The second group of bNAbs included 35O22, which targets an Env epitope in the gp120-gp41 interface (52), and 10-1074, which targets a gp120 glycan-dependent V3 epitope (53). These bNAbs showed no preference for a specific Env conformation and equivalently neutralized viruses with Envs altered in gp120 residue 193 (Fig. 4E).

The third group of bNAbs included PGT151, which targets an Env epitope in the gp120-gp41 interface (54), and three bNAbs directed against the gp41 membrane-proximal external region (MPER). All these bNAbs exhibited a trend favoring a state 2 conformation, but only PGT151 and 4E10 antibodies showed a statistically significant preference (Fig. 4E). This observation is consistent with previous suggestions that some MPER epitopes on gp41 are more exposed after CD4 binding (55, 56). Overall, we found that many naturally elicited antibodies, including several bNAbs, exhibit selectivity for specific Env conformations (see Table S4 in the supplemental material).

We next used the bNAbs with high conformational selectivity to study the effects of the L193A change in the gp120 Env of other HIV-1 strains. The L193A change in HIV-1 strains from clades A, B, and C led to phenotypes consistent with those expected for state 2 or 3 (Fig. 4F). Ligands preferring downstream Env conformations (sCD4 and 4E10) inhibited the L193A viruses more effectively than the related WT viruses. Conversely, ligands preferring state 1 (PG9 and VRC03) more potently inhibited the WT strains. Thus, Env residue 193 maintains Envs from different HIV-1 clades in state 1, countering transitions to downstream conformations. The high degree of conservation (98%) of leucine at this position among all HIV-1 strains is consistent with its key role in maintaining the integrity of state 1.

## DISCUSSION

Our report provides new insights into the relationship between the Env conformational landscape, virus entry requirements, and HIV-1 susceptibility to inhibitors and antibodies. Changes in specific gp120 V1/V2 residues resulted in increased resistance to BMS-806, a blocker of conformational change, and increased sensitivity to sCD4, CD4MCs, and CD4i, V2, and V3 antibodies. Significant increases in sensitivity to T20, reflecting formation/exposure of the gp41 HR1 coiled coil, were not observed for most of these mutants. Moreover, the mutants remained CD4 dependent. Thus, these mutants sample a set of related entry-compatible conformations that are intermediate between state 1 and the full CD4-bound state (state 3). smFRET analysis revealed that these Env mutants are enriched in the occupancy of state 2, thus linking this obligate intermediate state to phenotypically characterized, functional Env conformations on the HIV-1 entry pathway.

**FIG 5:**
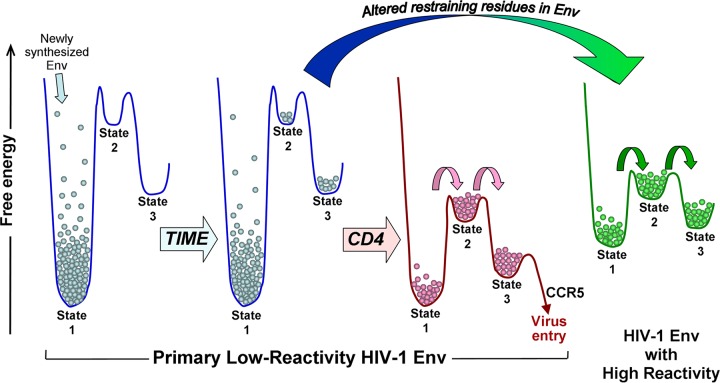
Model of HIV-1 Env conformational landscapes. Effects of time, CD4 binding, and alteration of restraining residues on the conformations occupied by a primary HIV-1 Env. Most primary HIV-1 Envs have a low propensity to change from state 1 (i.e., they have low Env reactivity [32]). Alteration of key restraining residues can convert an Env with low reactivity to a more reactive Env (large arrow). The conformational landscape of the unliganded high-reactivity Env resembles that of the CD4-bound low-reactivity Env. Small arrows indicate increased transition rates, relative to those of the unliganded low-reactivity Env.

The conformational states occupied by the functional HIV-1 Env trimer depend upon two related parameters: (i) the height of the activation barriers separating the states, which determines the rates of transitions between the states, and (ii) the relative free energies of the states, which dictate the occupancy of each state at equilibrium. The major fraction of primary HIV-1 Envs resides in state 1, with transitions from this metastable state constrained by the high activation barriers separating state 1 and state 2 (Fig. 5). Under certain circumstances, these high activation barriers may allow Env-receptor engagement or antibody binding to state 1 before equilibrium among the available conformations is achieved. Indeed, antibodies that potently neutralize primary HIV-1 isolates exhibit high rates of binding to Env trimers in state 1 (41, 57, 58). The Env energy landscape changes upon CD4 binding and upon alteration of specific conformation-restraining residues in gp120 (Fig. 5). In both cases, the activation barriers and the differences in free energy between state 1 and downstream conformations are lowered, increasing the propensity for the CD4-bound and mutant HIV-1 Envs to proceed along the entry pathway. Lowered activation barriers allow more transitions between states, and the smaller differences between the free energies of the states result in an increase in the occupancy of state 2 relative to that of state 1. Of note, multiple different Env residue changes resulted in qualitatively similar viral phenotypes; this observation favors a model in which changes in restraining Env residues destabilize state 1, allowing Env to proceed to state 2. Some of the Env changes may have fortuitously stabilized state 2 as well. State 2 encompasses a set of related conformations that reside in a local energy well. Previous studies suggested that the HIV-1 gp120 core has a propensity to sample the CD4-bound state when variable loop-mediated restraints are removed (59). In a similar manner, the more subtle losses of key molecular contacts resulting from single-residue changes in the gp120 trimer association domain release Env to assume one or more intermediate states. Importantly, Envs in state 2 retain a high potential energy, which likely is required for Env function; however, due to the low activation barrier between states 2 and 3, Envs in state 2 are more sensitive to triggering by ligands, including CD4 itself, that drive Env to the CD4-bound state. Destabilization of the ground state of a typical protein is expected to result in a decrease in function. In contrast, destabilization of state 1 creates increased opportunities for HIV-1 Env to sample downstream functional conformations. Smaller activation barriers and more-favorable free energy differences between state 1 and state 2 should allow a higher occupancy of Env protomers by CD4, lowering CD4 requirements for productive infection. CD4-independent HIV-1 may move even further along the entry pathway than the L193 mutants.

Alteration of several gp120 restraining residues, some of which exhibit variability in natural HIV-1 strains, can influence virus susceptibility to conformation-sensitive ligands. Hypersensitivity of the L193 Env mutants to these ligands reflects an increased sampling of state 2. We hypothesize that the observed variation in sensitivity of natural HIV-1 strains to conformation-sensitive ligands (32, 41, 60) results from altered activation barriers between state 1 and downstream conformations, either as a result of destabilization of state 1 or by changes more specific for the CD4 activation process. Viruses may thus achieve a balance between resistance to neutralization by host antibodies and the level of target cell CD4 required to trigger virus entry.

## MATERIALS AND METHODS

Detailed descriptions of the methods used are provided in Text S1 in the supplemental material.

### Viral infection assay.

A single-round infection assay was performed in 96-well plates by adding to each well a test compound or an antibody followed by supernatant containing a specific Env-pseudotyped virus (4 ng of p24) and then Cf2Th-CD4/CCR5 target cells. The activity of firefly luciferase, which was used as a reporter protein in the system, was measured after 48 h of incubation at 37°C. Values corresponding to 50% infectivity concentrations (IC_50_s) were calculated by fitting the data to the four-parameter (logistic) equation (37).

### Single-molecule fluorescence resonance energy transfer.

Analysis of the conformational dynamics of HIV-1 Env was done after enzymatic labeling of the V1 and V4 loops on native HIV-1 virions with Cy3 and Cy5 fluorophores, respectively (43).

### Flow cytometry.

Flow cytometric analysis was performed by incubating transfected 293T cells with various concentrations of a test compound or an antibody. Binding was detected with allophycocyanin-conjugated anti-human antibody and/or fluorescein isothiocyanate-conjugated anti-CD4 antibody and analyzed with a BD FACSCanto II flow cytometer (BD Biosciences).

## SUPPLEMENTAL MATERIAL

Text S1  Supplemental experimental procedures. Download Text S1, DOCX file, 0.1 MB

Figure S1  Interaction of small molecules with HIV-1 Env. (A) Deletion of the cytoplasmic tail of HIV-1 Env exposes the epitope of the 17b antibody. The binding of the 17b or 2G12 antibody to either HIV-1_JR-FL_ cytoplasmic tail-deleted Env (JR-FL ΔCT) or HIV-1_JR-FL_ full-length Env (JR-FL FL) was measured by flow cytometry. The increased exposure of CD4i epitopes as a result of truncation of the HIV-1 Env cytoplasmic tail has been previously reported (Wyss et al., 2005; Chen et al., 2015 [Text S1]). (B) The effect of either BMS-806 or DMJ-II-121 on the binding of the 17b and 2G12 antibodies to the gp120 Env captured by the D7324 antibody on enzyme-linked immunosorbent assay (ELISA) plates is shown. The effect of the compounds on the binding of the 17b antibody to the monomeric gp120 was similar to that observed for the cell-surface Env trimer (Fig. 1B). (C) The effect of BMS-806 on the sCD4-induced movement of the V1/V2 loop and the effect of DMJ-II-121 on V1/V2 conformation were detected by flow cytometry using the PG9 antibody. Download Figure S1, DOC file, 0.1 MB

Figure S2  Relationships between the sensitivities of viruses with Env amino acid changes at position 193 to different ligands. Each plot shows a pairwise comparison of the sensitivity (IC_50_) of each HIV-1 mutant to the specified ligands. Viruses have Envs with the indicated amino acid residue at position 193 in gp120 (wild-type HIV-1_JR-FL_ carries Leu at this position). The IC_50_ values of the 19b, 17b, and 902090 antibodies are reported as micrograms per milliliter (µg/ml), sCD4 and T20 as nanomoles per liter (nM), and polyclonal sera (PS) from HIV-1-infected individuals as the reciprocal of the dilution. Rs, Spearman’s Rho coefficient; (*P*), two-tailed *P* value. Significant correlations (*P* < 0.05) are shown with blue letters. Download Figure S2, DOC file, 0.2 MB

Figure S3  Sensitivity of HIV-1_JR-FL_ Env variants to the 830A Fab antibody fragment. (A) The 830A epitope was mapped on the clade C ZM109 scaffold (protein database entry 4YWG), with antibody contacts shown in red. (B) Sensitivity of wild-type HIV-1_JR-FL_ (red) and the specified HIV-1 Env mutants (light blue) to neutralization by the 830A Fab. Env variants that contain changes in the predicted epitope of 830A are labeled with an asterisk. Data are averages of results of two independent experiments and were fitted to the logistic (four-parameter) equation. Download Figure S3, DOC file, 0.3 MB

Figure S4  Survival curves for the transitions between specific states. Dwell time histograms were fitted to exponential distributions to estimate the rate constant for each transition. The calculated rates are shown in Table S3. Download Figure S4, DOC file, 0.6 MB

Figure S5  The relationship between antibody neutralization and combined sensitivity (overall sensitivity based on the combination of IC_50_s) of HIV-1 Env variants with changes in gp120 residue 193 to the different conformation-sensitive ligands. (A) Relationship between the hydrophobicity of gp120 residue 193 and the combined sensitivity of HIV-1_JR-FL_ variants to neutralization by conformation-sensitive Env ligands. Calculation of the combined sensitivity is based on the IC_50_ of each ligand as described in Text S1. (B) Relationship between the combined sensitivity of HIV-1_JR-FL_ variants and the neutralization of virus variants by antibodies found in the serum of HIV-1-infected individuals. (C and D) Data were determined as described for panel B, but the relationship was tested for CD4-BS antibodies (C) and various bNAbs (D). Spearman’s Rho coefficient and two-tailed *P* values are reported for each analysis. Data shown are averages of results obtained in two or three independent experiments. Download Figure S5, DOC file, 0.2 MB

Table S1  Phenotypes of HIV-1_JR-FL_ Env mutants. Table S1, DOC file, 0.1 MB

Table S2  Ligands used in the study. Table S2, DOC file, 0.1 MB

Table S3  Rate constants and free energy differences associated with the WT and L193 mutant HIV-1_JR-FL_ Envs, unliganded and after sCD4_D1D2_ binding. Table S3, DOC file, 0.1 MB

Table S4  Sensitivity of HIV-1 Env conformational states to inhibition by Env ligands. Table S4, DOC file, 0.1 MB
