# Supplementary material for: Release of gp120 Restraints Leads to an Entry-Competent Intermediate State of the HIV-1 Envelope Glycoproteins
Source: mBio. 2016 Oct 25;7(5):e01598-16. doi: 10.1128/mBio.01598-16 (PMC5080382; doi:10.1128/mBio.01598-16)
Supplement: Text S1 — Supplemental experimental procedures. Download [file mbo005163034s1.docx]

**SUPPLEMENTAL INFORMATION**

**Release of gp120 Restraints Leads to an Entry-Competent Intermediate State of the HIV-1 Envelope Glycoproteins**

Alon Herschhorn^a,b*^, Xiaochu Ma^c^, Christopher Gu^a^, John D. Ventura^c^**,** Luis Castillo-Menendez^a,b^, Bruno Melillo^d^, Daniel S. Terry^e^, Amos B. Smith III^d^, Scott C. Blanchard^e^, James B. Munro^f^, Walther Mothes^c^, Andres Finzi^g^ and Joseph G. Sodroski^a,b,h^*

**SUPPLEMENTAL EXPERIMENTAL PROCEDURES**

**Cell Lines**

293T cells were grown in Dulbecco’s Modified Eagle Medium (DMEM) containing 10% FBS, 100 µg/ml streptomycin and 100 units/ml penicillin. Cf2Th-CD4/CCR5 cells were grown in the same medium supplemented with 400 µg/ml G418 and 200 µg/ml hygromycin B (both from Invitrogen, Carlsbad, CA).

**HIV-1 Env mutants**

Mutations were introduced into the plasmid expressing the HIV-1_JR-FL_ Envs using the QuikChange II site-directed mutagenesis protocol, QuikChange multi site-directed mutagenesis kit (Stratagene) or PCR overlap assembly. The presence of the desired mutations was confirmed by DNA sequencing. All Env residues are numbered according to convention, based upon the HXBc2 prototypic sequence.

**Production of recombinant HIV-1 expressing luciferase**

Viruses were produced as previously described (Herschhorn et al., 2014). Briefly, 293T cells were cotransfected with an envelope-expression plasmid, pHIVec2.luc plasmid and psPAX2 plasmid (Cat# 11348, NIH AIDS Research and Reference Reagent Program) in a ratio of 1:6:3 using Effectene (Qiagen). After a 48-hour incubation, the cell supernatant was collected and centrifuged for 5 minutes at 600 x g at 4°C. The amount of p24 in the supernatant was measured using the HIV-1 p24 antigen capture assay (Cat# 5421, Advanced BioScience Laboratories) and the virus-containing supernatant was frozen at -80°C.

For infection of monocyte-derived macrophages, viruses were prepared as follows: viruses carrying wild-type, L193A, or L193R mutant HIV_JR-FL_ Env were generated by transfection of HEK293 cells at a 1:1 ratio of pNL4-3 ΔEnv and pCAAGS­_gp160_JR-FL_, pCAAGS­_gp160_JR-FL_L193A_, or pCAAGS­_gp160_JR-FL_L193R_ using Fugene 6 (Promega). Supernatants from 24 and 48 h post-transfection were combined, filtered through a 0.45 μM filter (Pall Corporation) and titered on TZMbl cells. Titers were determined 48 h post-infection either by measuring luciferase activity using the firefly luciferase assay kit (Promega) or by measuring intracellular p24 levels using cytofix/cytoperm permeabilzation solution fixative (BD Biosciences) and the anti-p24 antibody KC57-RD2 (Beckman Coulter).

**Viral Infection Assay**

Small-molecule inhibitors were tested in a 96-well B&W isoplate (PerkinElmer, Boston, MA) using an HP D300 Digital Dispenser as previously described (Herschhorn et al., 2014). DMSO was used as a control and the final volume of either diluted compound or DMSO was 450 nl. Eighty microliters of supernatant containing 4 ng of p24 of viruses pseudotyped with a specific Env was added to each well and incubated briefly at room temperature. Following incubation, fifty microliters of 1x10^5^ Cf2-CD4/CCR5 target cells was added to each well and the plate was incubated for 48 hours at 37°C. The medium was then aspirated and cells were lysed with 30 µl of Passive Lysis Buffer (Promega, cat# E1941). The activity of the firefly luciferase, which was used as a reporter protein in the system, was measured with a Centro LB 960 luminometer (Berthold Technologies, TN, USA). Peptides, antibodies and sCD4 were tested in a similar manner but they were first diluted in DMEM; thirty microliters of each tested concentration was manually dispensed in a 96-well plate and then thirty microliters of 4 ng of p24 of viruses pseudotyped with a specific Env was added. After a brief incubation at room temperature, thirty microliters of 1.7x10^5^ Cf2-CD4/CCR5 target cells was added. All other steps were performed as above.

To assess the effect of exposure to cold on virus infectivity, equal amounts of recombinant virus preparations (measured by reverse transcriptase activity) were incubated on ice for different periods of time. At the end of the incubation, aliquots were removed and transferred to a -80°C freezer until infection. The frozen aliquots were thawed at 37°C and added to target cells to assess infectivity.

Monocyte-derived macrophage (MDM) infection

MDMs were generated from PBMCs isolated using Ficoll-Paque Plus (GE Healthcare) following adherence plating for 2 h at 37°C in T75 filtered Poly-D-Lysine-coated flasks (Corning) in macrophage growth medium (RPMI 1640 supplemented with 10% fetal calf serum (FCS), 100 μg/ml streptomycin, 100 U/ml penicillin, and 2 mM glutamine, 1% Na-pyruvate, 1% Non-Essential Amino Acids). Suspension cells were removed, attached monocytes were washed twice with PBS, and macrophage growth medium was replaced with 10 ml macrophage differentiation medium (RPMI 1640 supplemented with 10% fetal calf serum (FCS), 100 μg/ml streptomycin, 100 U/ml penicillin, and 2 mM glutamine, 1% Na-pyruvate, 1% Non-Essential Amino Acids, and 50 ng/ml rhM-CSF (R&D Systems)) and allowed to differentiate for 1-2 weeks at 37°C. After 1-2 weeks, macrophages were removed with 5 ml Accutase cell detachment solution (Biolegend) at RT for 30 minutes. Macrophages were plated into 96-well flat bottom tissue-culture treated polystyrene plates at a density of 30,000 cells per 100 μl macrophage differentiation media per well. For infection, HIV-containing supernatant was concentrated with Lenti-X concentrator (Clonetech) according to the manufacturer’s instructions and resuspended in 50 μl macrophage differentiation media and added to each well containing MDMs for a total volume of 150 μL. Plates were spinoculated for 2 h at 1200 x g. Infections proceeded for 4 days and intracellular p24 levels were assessed using flow cytometry. Briefly, HIV-1-infected MDM populations were defined as p24 positive cells (antibody KC57-RD2, Beckman Coulter) gated to live CD14/CD71 double-positive populations using anti-CD14-FITC antibody HCD14 (Biolegend) and anti-CD71-APC antibody CY1G4 (Biolegend). Alternatively, recombinant firefly luciferase-expressing viruses were concentrated by ultracentrifugation (30,000 RPM, SW 41 Ti swinging-bucket rotor (Beckman Coulter) for 2 h at 4ºC). Virus titers were calculated on Affinofile cells that were induced for maximum expression of the CD4 and CCR5 receptors. Volumes of the WT and L193A mutant virus preparations with equivalent infectivity on Affinofile cells were used to infect MDMs. The cells were lysed 4 days after infection and the relative luciferase activity in the lysate was measured.

**Data Analysis**

All viral infection assays were done in duplicate. The readout of each duplicate was averaged and normalized to an internal control on the same plate (4-8 replicates) that included DMSO (for small-molecule inhibitors) or DMEM (for all other ligands) instead of the diluted test molecule. Results were fitted to the four-parameter (logistic) equation using the Graphpad Prism 6 program (GraphPad Software, Inc., CA, USA), after building the equation in the program.

Combined sensitivity indices were calculated according to the following equations:

FC (Ligand) = mutant IC_50_ / WT IC_50_

Combined sensitivity = FC(sCD4) x FC(19b) x FC(17b) x FC(902090) x FC(T20) x FC(DMJ-II-121)

where FC = fold change, Ligand = one of the following: sCD4, 19b, 17b, 902090, T20, or DMJ-II-121. For sCD4, FC= relative mutant binding*IC_50_ / WT IC_50_ where the binding of each Env variant to sCD4 was normalized to the response of WT and then used to normalize virus inhibition.

A statistical nonparametric analysis was done by a Spearman rank correlation test, using the Graphpad Prism 6 program. A summary of the data statistics is included for each analysis in the relevant figure legend.

Env structures were analyzed and displayed using the UCSF Chimera package (Pettersen et al., 2004).

**Immunoprecipitation and Analysis of Radiolabeled Envs**

Three hundred thousand 293T cells were transfected with the pcDNA3.1 vector expressing the codon-optimized HIV-1_JR-FL_ env gene using calcium phosphate. One day after transfection, cells were metabolically labeled for 16 h with 100 μCi/mL [35S]- methionine-cysteine ([35S] Protein Labeling Mix; Perkin-Elmer) in DMEM medium lacking methionine and cysteine and supplemented with 5% dialyzed fetal bovine serum.  Cells were subsequently lysed in RIPA buffer (140 mM NaCl, 8 mM Na_2_HPO_4_, 2 mM NaH_2_PO_4_, 1% NP40, 0.05% sodium dodecyl sulfate (SDS)).  Precipitation of radiolabeled HIV-1_JR-FL_ Envs from cell lysates or shed gp120 from the medium was performed with a mixture of sera from HIV-1-infected individuals for 1 hour at 4°C in the presence of 50 ul of 10% Protein A-Sepharose (American BioSciences). All samples were loaded on NuPAGE Novex Bis-Tris polyacrylamide gels (Invitrogen). Gels were analyzed by autoradiography and PhosphorImager (Molecular Dynamics) to assess the association between the gp41 and gp120 subunits and the efficiency of proteolytic cleavage, as previously described (Finzi et al., 2010).

**Single-Molecule Fluorescence Resonance Energy Transfer (smFRET)**

smFRET imaging was performed essentially as described (Munro et al., 2014). Briefly, short enzymatic labeling peptides Q3 (GQQQLG) and A1 (GDSLDMLEWSLM) (Lin et al., 2006; Zhou et al., 2007) were introduced into the V1 and V4 loops, respectively, of the wild-type and mutant HIV-1_JR-FL_ Env-expressing plasmids (pCAGGS_JR-FL_L193A or L193R). Viral particles were produced by co-transfecting HEK 293 cells with the backbone plasmid pNL4-3 ΔRT ΔEnv and the Env-expressing plasmids at a ratio of 1:1. The Env-expressing plasmids contained a mixture of tagged and untagged plasmids at 1:40 ratio. Viruses were harvested 40 h post-transfection, concentrated at 20,000 g, and labeled at room temperature overnight with 0.5 μM Cy3B(3S)-cadaverine using 0.65 μM transglutaminase (Sigma), and with 0.5 μM Cy5(4S)COT*-CoA (catalog number LD650, Lymidyne Technologies) using 5 μM AcpS. Viruses were incubated with 0.1 mg/ml DSPE-PEG_2000_-biotin (Avanti) for 30 min, purified using a 6%-18% Optiprep gradient with 50 mM Tris pH 7.4 and 50 mM NaCl, under 150,000 g for 1 h and stored at -80°C. For imaging, viral particles were immobilized on quartz slides with surface-coated streptavidin in imaging buffer (50 mM NaCl, 50 mM Tris pH 7.4) containing a cocktail of triplet-state quenchers (Dave et al., 2009), and an enzymatic oxygen scavenging system consisting of protocatachuate dioxygenase (PCD) and protocatachuic acid PCA (Aitken et al., 2008). Virus particles were imaged on a prism-based TIRF microscope equipped with a 1.27 NA 60 X water-immersion objective (Nikon) (Juette et al., 2016). The donor was excited with a 532-nm laser (Laser Quantum). The donor and acceptor signals were collected at 40-ms time resolution for 2000 frames using an sCMOS camera (Hamamatsu). For ligand binding assays, the surface immobilized virions were incubated with 0.1 mg/ml sCD4_D1D2_ for 30 min at room temperature prior to imaging. FRET trajectories were analyzed using customized MatLab scripts (Juette et al., 2016). FRET histograms were fit to the sum of three Gaussian distributions. FRET traces were idealized using Hidden Markov Modeling (HMM), and rate constants for transitions in FRET were determined by fitting dwell time histograms to exponential distributions (Figure S4). The ΔG and ΔΔG_ij_ were calculated by the following equations:

1. ΔG = -*k_B_T* ln(*P_i_/P_j_*)

where *P_i_* and *P_j_* are the occupancies of the ith and jth state in the histogram; and

2. *ΔΔG_ij_ = -k_B_T* ln*(k_ij_^sCD4^/k_ij_^unliganded^)*, or *ΔΔG_ij_ = -k_B_T* ln*(k_ij_^mutant^/k_ij_^WT^)*

where *k_ij_* is the rate of transition from the ith to jth FRET state.

The uncertainties in the ΔG and ΔΔG values were estimated by propagating the 95% confidence intervals from the histogram fitting into the energy calculations. FRET histograms and transition density plots of the WT HIV-1_JR-FL_ Env (Figure 3A), which were used as reference controls, were generated as part of a large project and will be published also in Ma et al., 2016.

**Flow Cytometry**

Plasmids expressing the wild-type HIV-1_JR-FL_, HIV-1_JR-FL_ΔCT or the double mutant HIV-1_JR-FL_ E168K/N188AΔCT Env were transfected with the Effectene transfection reagent (Qiagen) into 293T cells. After 48-72 hours, cells were detached with 5 mM EDTA/PBS and 0.2-1 million cells were briefly incubated with various concentrations of a test compound and then with or without indicated concentrations of sCD4. C34-Ig (at a final concentration of 20 μg/mL) or a specified antibody (at a final concentration of 1 μg/mL) was added to the cells. After a 30-minute incubation, the cells were washed twice and incubated with Allophycocyanin-conjugated F(ab′)2 fragment donkey anti-human IgG antibody (1:100 dilution; catalog no. 709-136-149; Jackson ImmunoResearch Laboratories) and Fluorescein isothiocyanate-conjugated anti-CD4 antibody (1:33 dilution, E-biosciences) for 15 minutes. Cells were washed twice and analyzed with a BD FACSCanto II flow cytometer (BD Biosciences). All procedures were performed at room temperature.

**REFERENCES (for Supplemental Experimental Procedures and Figures)**

Aitken, C.E., Marshall, R.A., and Puglisi, J.D. (2008). An oxygen scavenging system for improvement of dye stability in single-molecule fluorescence experiments. Biophys J. 94: 1826-35.

Blattner, C., Lee, J.H., Sliepen, K., Derking, R., Falkowska, E., de la Peña, A.T., Cupo, A., Julien, J.P., van Gils, M., Lee, P.S., Peng, W., Paulson, J.C., Poignard, P., Burton, D.R., Moore, J.P., Sanders, R.W., Wilson, I.A., Ward, A.B. (2014). Structural delineation of a quaternary, cleavage-dependent epitope at the gp41-gp120 interface on intact HIV-1 Env trimers. Immunity. *40*:669-80.

Cavacini, L.A., Emes, C.L., Power, J., Buchbinder, A., Zolla-Pazner, S., Posner, M.R. (1993). Human monoclonal antibodies to the V3 loop of HIV-1 gp120 mediate variable and distinct effects on binding and viral neutralization by a human monoclonal antibody to the CD4 binding site. J Acquir Immune Defic Syndr. *6*:353-8.

Chen, J., Kovacs, J.M., Peng, H., Rits-Volloch, S., Lu, J., Park, D., Zablowsky, E., Seaman, M.S., and Chen, B. (2015). HIV-1 ENVELOPE. Effect of the cytoplasmic domain on antigenic characteristics of HIV-1 envelope glycoprotein. Science *349*:191-5.

Dave, R., Terry, D.S., Munro, J.B., and Blanchard, S.C. (2009). Mitigating unwanted photophysical processes for improved single-molecule fluorescence imaging. Biophys. J. 96: 2371-81.

Finzi, A., Pacheco, B., Xiang, S.H., Pancera, M., Herschhorn, A., Wang, L., Zeng, X., Desormeaux, A., Kwong, P.D., and Sodroski, J. (2012). Lineage-specific differences between human and simian immunodeficiency virus regulation of gp120 trimer association and CD4 binding. J Virol *86*, 8974-8986.

Finzi, A., Xiang, S.H., Pacheco, B., Wang, L., Haight, J., Kassa, A., Danek, B., Pancera, M., Kwong, P.D., and Sodroski, J. (2010). Topological layers in the HIV-1 gp120 inner domain regulate gp41 interaction and CD4-triggered conformational transitions. Mol. Cell. *37*, 656-667.

Huang, J., Ofek, G., Laub, L., Louder, M.K., Doria-Rose, N.A., Longo, N.S., Imamichi, H., Bailer, R.T., Chakrabarti, B., Sharma, S.K., Alam, S.M., Wang, T., Yang, Y., Zhang, B., Migueles, S.A., Wyatt, R., Haynes, B.F., Kwong, P.D., Mascola, J.R., Connors, M. (2012). Broad and potent neutralization of HIV-1 by a gp41-specific human antibody. Nature *491*:406-12.

Huang, J., Kang, B.H., Pancera, M., Lee, J.H., Tong, T., Feng, Y., Imamichi, H., Georgiev, I.S., Chuang, G.Y., Druz, A., Doria-Rose, N.A., Laub, L., Sliepen, K., van Gils, M.J., de la Peña, A.T., Derking, R., Klasse, P.J., Migueles, S.A., Bailer, R.T., Alam, M., Pugach, P., Haynes, B.F., Wyatt, R.T., Sanders, R.W., Binley, J.M., Ward, A.B., Mascola, J.R., Kwong, P.D., Connors, M. (2014). Broad and potent HIV-1 neutralization by a human antibody that binds the gp41-gp120 interface. Nature. *515*:138-42.

Herschhorn, A., Gu, C., Espy, N., Richard, J., Finzi, A., and Sodroski, J.G. (2014). A broad HIV-1 inhibitor blocks envelope glycoprotein transitions critical for entry. Nat Chem Biol *10*, 845-852.

Juette, M.F., Terry, D.S., Wasserman, M.R., Altman, R.B., Zhou, Z., Zhao, H. and Blanchard, S.C. (2016). Single-molecule imaging of non-equilibirum molecular ensembles on the millisecond timescale. Nat. Methods, submitted.

Ma, X., Terry, D.S., Gorman, J., Hong, X., Arthos, J., Kwong, P.D., Blanchard, S.C, Mothes, M.*, and Munro, J.B.* (2016). Single-molecule FRET suggests asymmetric trimer as an intermediate during HIV-1 entry. In preparation

Munro, J.B., Gorman, J., Ma, X., Zhou, Z., Arthos, J., Burton, D.R., Koff, W.C., Courter, J.R., Smith, A.B., 3^rd^, Kwong, P.D., Blanchard, S.C., and Mothes, W. (2014). Conformational dynamics of single HIV-1 envelope trimers on the surface of native virions. Science *346*, 759-763.

Lin, C.W., and Ting A.Y. (2006). Transglutaminase-catalyzed site-specific conjugation of small-molecule probes to proteins in vitro and on the surface of living cells. J. Am. Chem. Soc. 128: 4542-3.

Pan, R., Gorny, M.K., Zolla-Pazner, S., and Kong, X.P. (2015). The V1V2 region of HIV-1 gp120 forms a five-stranded beta barrel. Journal of Virology *89*, 8003-8010.

Pettersen, E.F., Goddard, T.D., Huang, C.C., Couch, G.S., Greenblatt, D.M., Meng, E.C., and Ferrin, T.E. (2004). UCSF Chimera--a visualization system for exploratory research and analysis. Journal of computational chemistry *25*, 1605-1612.

Posner, M.R., Cavacini, L.A., Emes, C.L., Power, J., Byrn, R.*J.* (1993). Neutralization of HIV-1 by F105, a human monoclonal antibody to the CD4 binding site of gp120. Acquired Immune. Defic. Syndr. *6*:7-14.

Rizzuto, C.D., Wyatt, R., Hernandez-Ramos, N., Sun, Y., Kwong, P.D., Hendrickson, W.A., and Sodroski, J. (1998). A conserved HIV gp120 glycoprotein structure involved in chemokine receptor binding. Science *280*, 1949-1953.

Scott, C.F., Jr., Silver, S., Profy, A.T., Putney, S.D., Langlois, A., Weinhold, K., and Robinson, J.E. (1990). Human monoclonal antibody that recognizes the V3 region of human immunodeficiency virus gp120 and neutralizes the human T-lymphotropic virus type IIIMN strain. Proceedings of the National Academy of Sciences of the United States of America *87*, 8597-8601.

Shingai, M., Nishimura, Y., Klein, F., Mouquet, H., Donau, O.K., Plishka, R., Buckler-White, A., Seaman, M., Piatak, M. Jr, Lifson, J.D., Dimitrov, D.S., Nussenzweig, M.C., Martin, M.A. (2013). Antibody-mediated immunotherapy of macaques chronically infected with SHIV suppresses viraemia. Nature. *503*:277-80.

Stiegler, G., Kunert, R., Purtscher, M., Wolbank, S., Voglauer, R., Steindl, F., Katinger, H. (2001). A potent cross-clade neutralizing human monoclonal antibody against a novel epitope on gp41 of human immunodeficiency virus type 1. AIDS Res. Human Retroviruses *17*:1757-1765.

Walker, L.M., Phogat, S.K., Chan-Hui, P.Y., Wagner, D., Phung, P., Goss, J.L., Wrin, T., Simek, M.D., Fling, S., Mitcham, J.L.*, et al.* (2009). Broad and potent neutralizing antibodies from an African donor reveal a new HIV-1 vaccine target. Science *326*, 285-289.

Wiehe, K., Easterhoff, D., Luo, K., Nicely, N.I., Bradley, T., Jaeger, F.H., Dennison, S.M., Zhang, R., Lloyd, K.E., Stolarchuk, C., *et al*. (2014). Antibody light-chain-restricted recognition of the site of immune pressure in the RV144 HIV-1 vaccine trial is phylogenetically conserved. Immunity *41*, 909-918.

Wu, X., Yang, Z.Y., Li, Y., Hogerkorp, C.M., Schief, W.R., Seaman, M.S., Zhou, T., Schmidt, S.D., Wu, L., Xu, L., Longo, N.S., McKee, K., O'Dell, S., Louder, M.K., Wycuff, D.L., Feng, Y., Nason, M., Doria-Rose, N., Connors, M., Kwong, P.D., Roederer, M., Wyatt, R.T., Nabel, G.J., Mascola, J.R. (2010). Rational design of envelope identifies broadly neutralizing human monoclonal antibodies to HIV-1. Science *329*:856-61.

Wyss, S., Dimitrov, A.S., Baribaud, F., Edwards, T.G., Blumenthal, R., and Hoxie, J. (2005). Regulation of human immunodeficiency virus type 1 envelope glycoprotein fusion by a membrane-interactive domain in the gp41 cytoplasmic tail. J. Virol. *79*:12231-41.

Zhou, Z., Cironi, P., Lin, A.J., Xu, Y., Hrvatin, S., Golan, D.E., Silver, P.A., Walsh, C.T., and Yin, J. (2007). Genetically encoded short peptide tags for orthogonal protein labeling by Sfp and AcpS phosphopantetheinyl transferases. ACS Chem. Biol. 2: 337-46.
