# Supplementary material for: Release of gp120 Restraints Leads to an Entry-Competent Intermediate State of the HIV-1 Envelope Glycoproteins
Source: mBio. 2016 Oct 25;7(5):e01598-16. doi: 10.1128/mBio.01598-16 (PMC5080382; doi:10.1128/mBio.01598-16)
Supplement: Figure S1 — Interaction of small molecules with HIV-1 Env. (A) Deletion of the cytoplasmic tail of HIV-1 Env exposes the epitope of the 17b antibody. The binding of the 17b or 2G12 antibody to either HIV-1JR-FL cytoplasmic tail-deleted Env (JR-FL ΔCT) or HIV-1JR-FL full-length Env (JR-FL FL) was measured by flow cytometry. The increased exposure of CD4i epitopes as a result of truncation of the HIV-1 Env cytoplasmic tail has been previously reported (Wyss et al., 2005; Chen et al., 2015 [Text S1]). (B) The effect of either BMS-806 or DMJ-II-121 on the binding of the 17b and 2G12 antibodies to the gp120 Env captured by the D7324 antibody on enzyme-linked immunosorbent assay (ELISA) plates is shown. The effect of the compounds on the binding of the 17b antibody to the monomeric gp120 was similar to that observed for the cell-surface Env trimer (Fig. 1B). (C) The effect of BMS-806 on the sCD4-induced movement of the V1/V2 loop and the effect of DMJ-II-121 on V1/V2 conformation were detected by flow cytometry using the PG9 antibody. Download [file mbo005163034sf1.doc]

A

B

Binding to soluble JR-FL gp120

17b binding (RLU)

2G12 binding (RLU)

17b binding (RLU)

2G12 binding (RLU)

JR-FL ΔCT

Control 17b 2G12

*P* = 0.004

*P* = 0.01


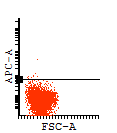

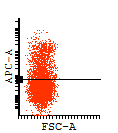

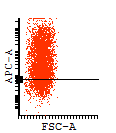


0.2%

0.3%

27.8%

81.8%

16.0%

Antibody binding (APC)

JR-FL FL


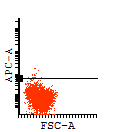

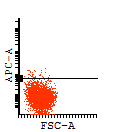

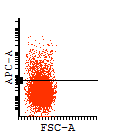


Forward scatter

0.8%

C


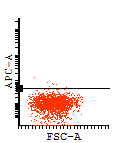

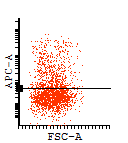

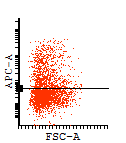

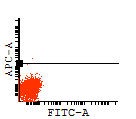

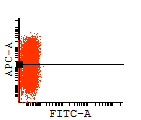

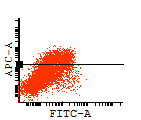


PG9 binding (APC)

sCD4

Forward scatter

DMJ-II-121

0.3%

31.1%

36.1%

Control

0%

24.6%

25.5%

BMS-806

Control

42.2%


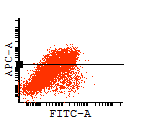


sCD4 binding (FITC)

**Figure S1. Interaction of small molecules with HIV-1 Env**

(A) Deletion of the cytoplasmic tail of HIV-1 Env exposes the epitope of the 17b antibody. The binding of the 17b or 2G12 antibody to either HIV-1_JR-FL_ cytoplasmic tail-deleted Env (JR-FL ΔCT) or HIV-1_JR-FL_ full-length Env (JR-FL FL) was measured by flow cytometry. The increased exposure of CD4i epitopes as a result of truncation of the HIV-1 Env cytoplasmic tail has been previously reported (Wyss et al., 2005; Chen et al., 2015). (B) The effect of either BMS-806 or DMJ-II-121 on the binding of the 17b and 2G12 antibodies to the gp120 Env captured by the D7324 antibody on ELISA plates is shown. The effect of the compounds on the binding of the 17b antibody to the monomeric gp120 is similar to that observed for the cell-surface Env trimer (Figure 1B). (C) The effect of BMS-806 on the sCD4-induced movement of the V1/V2 loop and the effect of DMJ-II-121 on V1/V2 conformation was detected by flow cytometry using the PG9 antibody.
