# Supplementary material for: Release of gp120 Restraints Leads to an Entry-Competent Intermediate State of the HIV-1 Envelope Glycoproteins
Source: mBio. 2016 Oct 25;7(5):e01598-16. doi: 10.1128/mBio.01598-16 (PMC5080382; doi:10.1128/mBio.01598-16)
Supplement: Figure S2 — Relationships between the sensitivities of viruses with Env amino acid changes at position 193 to different ligands. Each plot shows a pairwise comparison of the sensitivity (IC50) of each HIV-1 mutant to the specified ligands. Viruses have Envs with the indicated amino acid residue at position 193 in gp120 (wild-type HIV-1JR-FL carries Leu at this position). The IC50 values of the 19b, 17b, and 902090 antibodies are reported as micrograms per milliliter (µg/ml), sCD4 and T20 as nanomoles per liter (nM), and polyclonal sera (PS) from HIV-1-infected individuals as the reciprocal of the dilution. Rs, Spearman’s Rho coefficient; (P), two-tailed P value. Significant correlations (P < 0.05) are shown with blue letters. Download [file mbo005163034sf2.doc]

sCD4

R_s_ = 0.55 *P* = 0.017

R_s_ = 0.67 *P* = 0.023

R_s_ = 0.53 *P* = 0.023

R_s_ = 0.13 *P* = 0.61

R_s_ = 0.54 *P* = 0.026

R_s_ = 0.63 *P* =0.009

R_s_ = 0.74 *P* = 0.0004

R_s_ = 0.92 *P* < 0.0001

R_s_ = 0.84 *P* < 0.0001

R_s_ = 0.80 *P* = 0.0002

R_s_ = 0.33 *P* = 0.18

19b

R_s_ = 0.69 *P* = 0.0014

R_s_ = 0.22 *P* = 0.39

R_s_ = 0.87 *P* < 0.0001

R_s_ = 0.84 *P* < 0.0001

17b

R_s_ = 0.45 *P* = 0.063

R_s_ = 0.80 *P* = 0.0002

R_s_ = 0.76 *P* = 0.0006

902090

R_s_ = 0.07 *P* = 0.78

R_s_ = 0.11 *P* = 0.66

T20

19b

17b

902090

T20

PS1

PS2

**Figure S2. Relationships Between the Sensitivities of Viruses with Env Amino Acid Changes at Position 193 to Different Ligands**

Each plot shows a pairwise comparison of the sensitivity (IC_50_) of each HIV-1 mutant to the specified ligands. Viruses have Envs with the indicated amino acid residue at position 193 in gp120 (Wild-type HIV-1_JR-FL_ carries Leu at this position). The IC_50_ values of the 19b, 17b and 902090 antibodies are reported as μg/ml, sCD4 and T20 as nM, and polyclonal sera (PS) from HIV-1-infected individuals as the reciprocal of the dilution. R_s_, spearman’s Rho coefficient; *P*, two-tailed *P* value. Significant correlations (*P*<0.05) are shown with blue letters.
