# Supplementary material for: Release of gp120 Restraints Leads to an Entry-Competent Intermediate State of the HIV-1 Envelope Glycoproteins
Source: mBio. 2016 Oct 25;7(5):e01598-16. doi: 10.1128/mBio.01598-16 (PMC5080382; doi:10.1128/mBio.01598-16)
Supplement: Figure S3 — Sensitivity of HIV-1JR-FL Env variants to the 830A Fab antibody fragment. (A) The 830A epitope was mapped on the clade C ZM109 scaffold (protein database entry 4YWG), with antibody contacts shown in red. (B) Sensitivity of wild-type HIV-1JR-FL (red) and the specified HIV-1 Env mutants (light blue) to neutralization by the 830A Fab. Env variants that contain changes in the predicted epitope of 830A are labeled with an asterisk. Data are averages of results of two independent experiments and were fitted to the logistic (four-parameter) equation. Download [file mbo005163034sf3.doc]

**
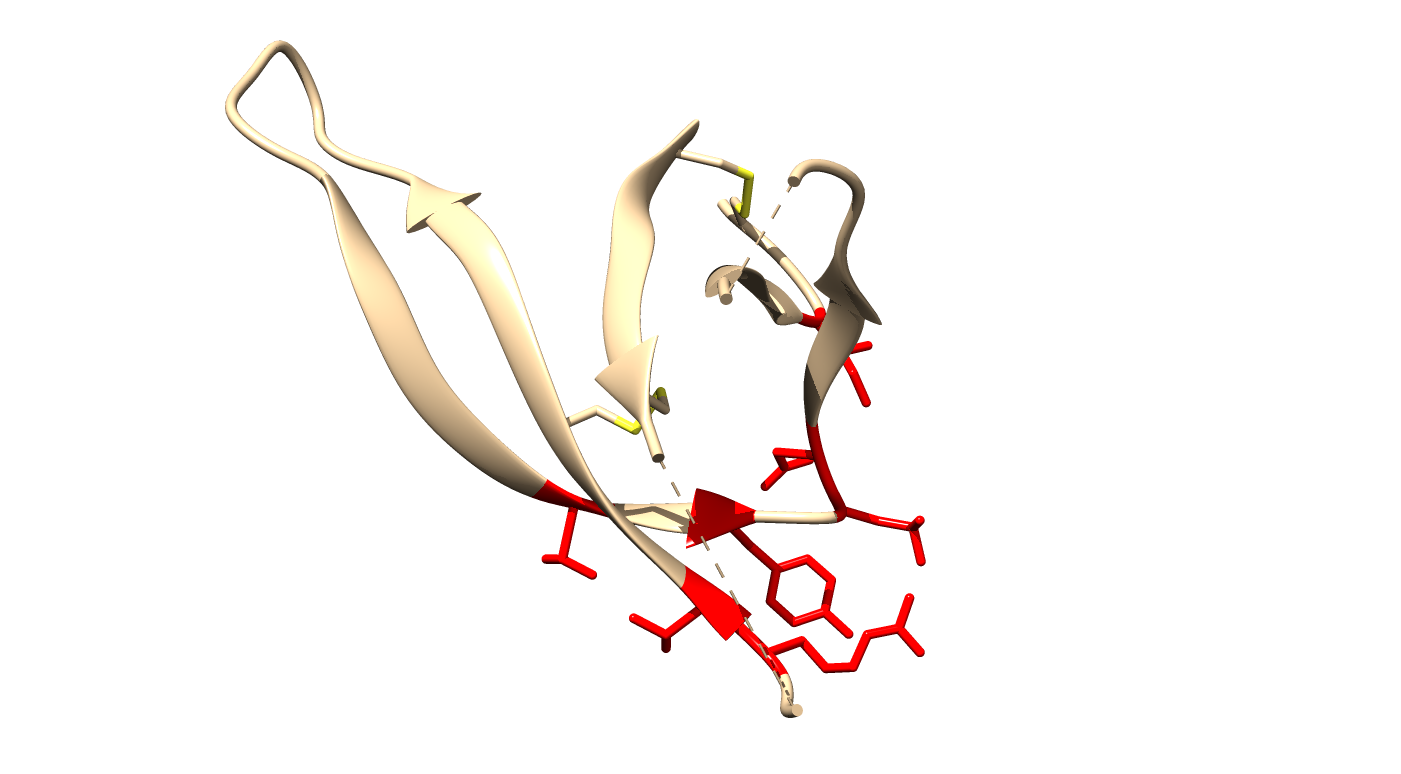
**

A

Ile 194

Asp 180

Leu 179

Thr 175

Arg 153

Val 154

Tyr 177

B

L193A Y191A Y177A* L175A*

Residual infection (%)

IC_50_ = 0.74 ± 0.13 μg/ml IC_50_ = 20.11 ± 7.24 μg/ml IC_50_ > 50 μg/ml IC_50_ = 21.49 ± 2.00 μg/ml

830 (μg/ml)

**Figure S3. Sensitivity of HIV-1_JR-FL_ Env variants to the 830A Fab antibody**

(A) The 830A epitope was mapped on the clade C ZM109 scaffold (Protein data base entry 4YWG), with antibody contacts shown in red. (B) Sensitivity of wild-type HIV-1_JR-FL_ (red) and the specified HIV-1 Env mutants (light blue) to the 830A Fab. Env variants that contain changes in the predicted epitope of 830A are labeled with an asterisk. Results are the average of two independent experiments and were fitted to the logistic (four-parameter) equation.
