# Supplementary material for: Release of gp120 Restraints Leads to an Entry-Competent Intermediate State of the HIV-1 Envelope Glycoproteins
Source: mBio. 2016 Oct 25;7(5):e01598-16. doi: 10.1128/mBio.01598-16 (PMC5080382; doi:10.1128/mBio.01598-16)
Supplement: Figure S4 — Survival curves for the transitions between specific states. Dwell time histograms were fitted to exponential distributions to estimate the rate constant for each transition. The calculated rates are shown in Table S3. Download [file mbo005163034sf4.doc]

**Figure S4. Survival Curves for the Transitions between Specific States**

Dwell time histograms were fitted to exponential distributions to estimate the rate constant for each transition. The calculated rates are shown in Table S4.
