# Supplementary material for: Release of gp120 Restraints Leads to an Entry-Competent Intermediate State of the HIV-1 Envelope Glycoproteins
Source: mBio. 2016 Oct 25;7(5):e01598-16. doi: 10.1128/mBio.01598-16 (PMC5080382; doi:10.1128/mBio.01598-16)
Supplement: Figure S5 — The relationship between antibody neutralization and combined sensitivity (overall sensitivity based on the combination of IC50s) of HIV-1 Env variants with changes in gp120 residue 193 to the different conformation-sensitive ligands. (A) Relationship between the hydrophobicity of gp120 residue 193 and the combined sensitivity of HIV-1JR-FL variants to neutralization by conformation-sensitive Env ligands. Calculation of the combined sensitivity is based on the IC50 of each ligand as described in Text S1. (B) Relationship between the combined sensitivity of HIV-1JR-FL variants and the neutralization of virus variants by antibodies found in the serum of HIV-1-infected individuals. (C and D) Data were determined as described for panel B, but the relationship was tested for CD4-BS antibodies (C) and various bNAbs (D). Spearman’s Rho coefficient and two-tailed P values are reported for each analysis. Data shown are averages of results obtained in two or three independent experiments. Download [file mbo005163034sf5.doc]

Combined sensitivity

Combined sensitivity

R_s_ = 0.65 *P* = 0.004

A

Patient 2 serum (1/titer_50_)

R_s_ = 0.77 *P* = 0.0006

B

R_s_ = 0.76 *P* = 0.0002

Patient 1 serum (1/titer_50_)

Hydrophobicity

VRC01

R_s_ = -0.67 *P* = 0.027

C

VRC03

R_s_ = -0.91 *P* < 0.0001

F105

R_s_ = 0.78 *P* = 0.004

Combined sensitivity

3BNC117

R_s_ = -0.88 *P* = 0.002

IC50 (μg/ml)

D

35O22

R_s_ = -0.08 *P* = 0.92

IC50 (μg/ml)

10-1074

R_s_ = 0.45 *P* = 0.27

PGT151

R_s_ = 0.62 *P* = 0.086

7H6

R_s_ = 0.51 *P* = 0.11

4E10

R_s_ = 0.63 *P* = 0.033

10E8

R_s_ = 0.33 *P* = 0.33

Combined sensitivity

**Figure S5. The Relationship Between Antibody Neutralization and the Combined Sensitivity (overall sensitivity based on the combination of IC50s) of HIV-1 Env Variants with Changes in gp120 Residue 193 to the Different Conformational Sensitive Ligands**

(A) Relationship between the hydrophobicity of gp120 residue 193 and the combined sensitivity of HIV-1_JR-FL_ variants to neutralization by conformation-sensitive Env ligands. Calculation of the combined sensitivity is based on IC_50_ of each ligand and described under the Supplemental Experimental Procedures. (B) Relationship between combined sensitivity of HIV-1_JR-FL_ variants and the neutralization of virus variants by antibodies found in the serum of HIV-1-infected individuals. (C,D) Same as B but the relationship was tested for CD4-BS antibodies (C) and various bNAbs (D). Spearman's Rho coefficient and two-tailed P values are reported for each analysis. Results shown are the average of those obtained in two or three independent experiments.
