# Supplementary material for: Release of gp120 Restraints Leads to an Entry-Competent Intermediate State of the HIV-1 Envelope Glycoproteins
Source: mBio. 2016 Oct 25;7(5):e01598-16. doi: 10.1128/mBio.01598-16 (PMC5080382; doi:10.1128/mBio.01598-16)
Supplement: Table S1 — Phenotypes of HIV-1JR-FL Env mutants. [file mbo005163034st1.doc]

**Table S1. Phenotypes of HIV-1_JR-FL_ Env Mutants**

| **HIV-1_JR-FL_ Env Variant^a^** | **gp120 region** | **Conserv-ation^b^** | **Association Index^c^** | **Processing Index^c^** | **Cell-Cell Fusion^c^** | **Relative Infectivity^c^** | **Relative sCD4 Sensitivity^d^** | **Cold-Inactivation^d^** |
| --- | --- | --- | --- | --- | --- | --- | --- | --- |
| WT |  |  | 1 | 1 | 1 | 1 | 1 | >48 |
| **V127A** | **V1** | **Ch Con** | **0.50** | **0.38** | **1.16** | **0.81** | **0.1** | **24** |
| L129A | V1 | Ch Con | 0.16 | 0.30 | 0.50 | 0.01 | 0.5 | 16 |
| V134A | V1 | Low | 1.18 | 0.97 | 0.93 | 2.16 | 0.2 | >48 |
| N136A | V1 | Low | 1.18 | 1.05 | 0.99 | 1.40 | 0.4 | >48 |
| N139A | V1 | Low | 1.17 | 1.54 | 0.75 | 1.00 | 0.5 | >48 |
| N141A | V1 | Low | 1.29 | 1.32 | 1.18 | 1.94 | 0.5 | >48 |
| ST143-  146AA | V1 | Low | 1.15 | 0.81 | 1.04 | 0.82 | 1 | >48 |
| M147A | V1 | Low | 1.13 | 0.99 | 0.78 | 1.23 | 0.2 | >48 |
| E153A | V1 | Low | 1.62 | 1.59 | 1.06 | 1.08 | 0.25 | >48 |
| **I154A** | **V1** | **Mod** | **0.93** | **0.96** | **0.50** | **1.55** | **0.06** | **24** |
| K155A | V1 | Mod | 1.21 | 1.56 | 0.95 | 1.21 | 0.2 | >48 |
| **N156A** | **V1** | **High** | **0.71** | **0.55** | **0.88** | **0.59** | **0.05** | **8** |
| F159A | V2 | Ch Con | 0.59 | 0.97 | 1.03 | 0.22 | 0.05 | 8 |
| N160A | V2 | Mod | 0.65 | 0.68 | 0.74 | 0.98 | 0.9 | >48 |
| TS163-  164AA | V2 | High-Low | 0.44 | 0.74 | 0.87 | 0.39 | >1 | >48 |
| I165A | V2 | Mod | 1.25 | 1.29 | 0.80 | 2.00 | 0.5 | >48 |
| R166A | V2 | Mod | 1.08 | 1.41 | 0.58 | 1.03 | 1 | >48 |
| D167A | V2 | High (PIV) | 0.49 | 0.67 | 1.44 | 0.78 | 0.5 | >48 |
| E168A | V2 | Mod | 0.82 | 1.07 | 0.93 | 0.90 | >1 | >48 |
| K171A | V2 | Mod | 1.36 | 1.70 | 0.63 | 0.68 | 0.65 | >48 |
| Y173A | V2 | Low | 1.35 | 2.21 | 0.81 | 1.28 | 0.4 | >48 |
| A174G | V2 | High (PIV) | 0.97 | 1.57 | nd | 0.74 | 1 | >48 |
| **L175A** | **V2** | **Mod** | **1.00** | **1.22** | **0.66** | **1.78** | **0.05** | **32** |
| F176A | V2 | Ch Con | 0.25 | 0.33 | 0.34 | << 0.01 | nd | nd |
| **Y177A** | **V2** | **Mod** | **0.64** | **2.28** | **0.88** | **2.04** | **0.09** | **40** |
| K178A | V2 | Mod | 1.10 | 1.24 | 0.24 | 0.58 | 0.5 | >48 |
| **L179G** | **V2** | **Mod** | **1.11** | **1.53** | **1.10** | 1.13 | **0.5** | **32** |
| D180A | V2 | Ch Con | 0.88 | 0.79 | 0.24 | 0.20 | 0.05 | >48 |
| V181G | V2 | Ch Con | 0.13 | 0.21 | 0.43 | 0.01 | nd | 8 |
| V182G | V2 | Mod | 0.82 | 0.63 | 0.54 | 0.45 | 0.1 | >48 |
| P183A | V2 | Mod | 1.21 | 0.99 | 1.52 | 0.95 | 0.4 | >48 |
| I184A | V2 | High | 0.25 | 0.74 | nd | 0.49 | 0.2 | >48 |
| D185A | V2 | Low | 0.85 | 1.57 | 0.81 | 0.63 | 0.5 | >48 |
| N187A | V2 | Low | 1.20 | 1.76 | 0.91 | 1.31 | 0.7 | >48 |
| N188A | V2 | Low | 0.95 | 1.54 | 0.83 | 1.34 | 0.7 | >48 |
| NN-187-  188AA | V2 | Low | 0.54 | 1.44 | 0.89 | 0.37 | 0.5 | >48 |
| **Y191A** | **V2** | **High** | **0.98** | **0.55** | **0.76** | **0.43** | **0.05** | **32** |
| R192A | V2 | Mod | 0.44 | 0.66 | 0.54 | 0.38 | 0.2 | >48 |
| **L193A** | **V2** | **High**  **Ch Con** | **0.46** | **0.72** | **0.63** | **0.38** | **0.1** | **8** |
| I194A | V2 | Mod | 0.93 | 1.51 | 0.69 | 0.46 | >1 | >48 |

**^a^**Env variants in bold were selected as potential candidates for Envs with increased reactivity (increased propensity to change conformation from State 1).

**^b^**The degree of conservation of the amino acid residue is indicated: High (PIV): conserved among primate immunodeficiency viruses; Ch Con: amino acid character conserved among primate immunodeficiency viruses; High: conserved among HIV-1 strains; Mod: moderate variation among HIV-1 strains; and Low: significant variation among HIV-1 strains.

**^c^**The association index, processing index, relative infectivity and cell-cell fusion activity were determined as described in the Supplemental Experimental Procedures. The values relative to those observed for the wild-type HIV-1_JR-FL_ Env are reported. These values represent the means of those obtained in at least three independent experiments, and typically vary no more than 20% among experiments. nd – not determined.

**^d^**The sensitivity of recombinant luciferase-expressing HIV-1 with the indicated HIV-1_JR-FL_ Env variants to cold incubation and to sCD4 was determined as described in the Supplemental Experimental Procedures. The relative sCD4 sensitivity represents the ratio of the mutant IC_50_ to wt IC_50_. The values for cold inactivation represent the half-lives in hours of virus infectivity following incubation on ice. The indicated values represent the means from at least three representative experiments, with variation of no more than 30% among experiments. nd – not determined.
