# Supplementary material for: Release of gp120 Restraints Leads to an Entry-Competent Intermediate State of the HIV-1 Envelope Glycoproteins
Source: mBio. 2016 Oct 25;7(5):e01598-16. doi: 10.1128/mBio.01598-16 (PMC5080382; doi:10.1128/mBio.01598-16)
Supplement: Table S2 — Ligands used in the study. [file mbo005163034st2.doc]

**Table S2. Ligands Used in the Study**

| **Ligand** | **Group** | **Type** | **Target** |
| --- | --- | --- | --- |
| sCD4 |  | Soluble receptor | gp120 CD4-binding site |
| 19b | Ligands that recognize CD4-bound Env conformations | Antibody | gp120 V3 loop (Scott *et al*., 1990) |
| 17b |  | Antibody | A discontinuous epitope that contains residues of the β2, β3, β20, and β21 sheets (Rizzuto *et al*., 1998) |
| 902090 |  | Antibody | A linear epitope composed of residues 171-177 (Wiehe *et al*., 2014) |
| 830A |  | Antibody | V2i (V2-integrin) antibody, contacts residues R153, V154, T175, Y177, L179, D180 and I194 on the surface of a gp120 V2 β-barrel (Pan *et al*., 2015 and see Figure S3) |
| T20 |  | Peptide | 36-amino acid peptide derived from gp41 HR2; binds gp41 HR1 coiled coil |
| F105 | Weakly neutralizing | Antibody | Weakly neutralizing CD4-binding site antibody (Posner et al., 1993; Cavacini *et al*., 1993) |
| VRC01  VRC03  3BNC117 | broadly neutralizing antibodies (bNAbs) | Antibody | CD4-binding site (Wu *et al*. 2010; Shingai *et al.*, 2013) |
| PG9 |  | Antibody | Quaternary V2 gp120 epitope (Walker *et al.*, 2009) |
| 35O22  PGT151 |  | Antibody | gp120-gp41 hybrid epitope (Huang *et al*., 2014; Blattner *et al.*, 2014) |
| 10-1074 |  | Antibody | V3-directed antibody; binding depends on the presence of glycosylation at Asn 332 (Shingai *et al.*, 2013) |
| 10E8  7H6  4E10 |  | Antibody | Membrane-proximal external region of gp41 (MPER) (Huang *et al*., 2012; Stiegler *et al*., 2001) |
