# Supplementary material for: Release of gp120 Restraints Leads to an Entry-Competent Intermediate State of the HIV-1 Envelope Glycoproteins
Source: mBio. 2016 Oct 25;7(5):e01598-16. doi: 10.1128/mBio.01598-16 (PMC5080382; doi:10.1128/mBio.01598-16)
Supplement: Table S3 — Rate constants and free energy differences associated with the WT and L193 mutant HIV-1JR-FL Envs, unliganded and after sCD4D1D2 binding. [file mbo005163034st3.doc]

**Table S3. Rate Constants and Free Energy Differences Associated with the WT and L193 Mutant HIV-1_JR-FL_ Envs, Unliganded and After sCD4_D1D2_ Binding**

| ***RATE CONSTANTS*** | | | | |
| --- | --- | --- | --- | --- |
|  | ***k*_1à2_ (s^-1^)** | ***k*_2à1_ (s^-1^)** | ***k*_2à3_ (s^-1^)** | ***k*_3à2_ (s^-1^)** |
| **WT HIV-1_JR-FL_** |  |  |  |  |
| Unliganded | 1.30±0.03 | 2.21±0.03 | 1.70±0.02 | 1.25±0.02 |
| sCD4 | 1.52±0.03 | 1.76±0.04 | 2.25±0.03 | 1.37±0.05 |
| **L193A HIV-1_JR-FL_** |  |  |  |  |
| Unliganded | 1.58±0.05 | 2.73±0.03 | 1.88±0.06 | 1.44±0.02 |
| sCD4 | 1.58±0.09 | 2.26±0.06 | 2.11±0.03 | 1.38±0.03 |
| **L193R HIV-1_JR-FL_** |  |  |  |  |
| Unliganded | 1.77±0.1 | 2.36±0.05 | 2.34±0.05 | 1.87±0.04 |
| sCD4 | 1.94±0.07 | 2.20±0.09 | 1.73±0.04 | 1.63±0.04 |

| ***FREE ENERGY DIFFERENCES*** | | | | |
| --- | --- | --- | --- | --- |
|  | ***ddG*_1à2_** | ***ddG*_2à1_** | ***ddG*_2à3_** | ***ddG*_3à2_** |
| **WT HIV-1_JR-FL_** |  |  |  |  |
| Unliganded/sCD4 | -0.16±0.03 | 0.23±0.02 | -0.28±0.02 | -0.09±0.04 |
| Unliganded WT/L193A | -0.19±0.04 | -0.21±0.01 | -0.1±0.03 | -0.14±0.02 |
| Unliganded WT/L193R | -0.31±0.06 | -0.07±0.03 | -0.32±0.03 | -0.40±0.03 |
| **L193A HIV-1_JR-FL_** |  |  |  |  |
| Unliganded/sCD4 | 0.001±0.06 | 0.19±0.02 | -0.12±0.03 | 0.04±0.02 |
| **L193R HIV-1_JR-FL_** |  |  |  |  |
| Unliganded/sCD4 | -0.09±0.07 | 0.07±0.05 | 0.30±0.03 | 0.13±0.03 |
