# Supplementary material for: Release of gp120 Restraints Leads to an Entry-Competent Intermediate State of the HIV-1 Envelope Glycoproteins
Source: mBio. 2016 Oct 25;7(5):e01598-16. doi: 10.1128/mBio.01598-16 (PMC5080382; doi:10.1128/mBio.01598-16)
Supplement: Table S4 — Sensitivity of HIV-1 Env conformational states to inhibition by Env ligands. [file mbo005163034st4.doc]

**Table S4. Sensitivity of HIV-1 Env Conformational States to Inhibition by Env Ligands**

| **Ligand** | **Env target** | **State 1** | **State 2** | **State 3** |
| --- | --- | --- | --- | --- |
| sCD4 | gp120 CD4-binding site | Relatively resistant | Relatively sensitive | Relatively sensitive |
| CD4-mimetic compounds (e.g., DMJ-II-121) | gp120 Phe 43 cavity (CD4-binding site) | Relatively resistant | Relatively sensitive | Relatively sensitive |
| Small-molecule conformational blockers  (e.g., BMS-806) | gp120 β20-β21 | Relatively sensitive | Relatively resistant | Relatively resistant |
| 902090 MAb | gp120 V2 (171-177) | Resistant | Sensitive | Sensitive |
| 830A Fab | Discontinuous gp120 V2i epitope on V2 β-barrel | Resistant | Sensitive | Sensitive |
| 19b MAb | gp120 V3 region | Resistant | Sensitive | Sensitive |
| 17b MAb | gp120 CD4i epitope | Resistant | Sensitive | Sensitive |
| PG9 bNAb | Quaternary gp120 V2 | Sensitive | Less sensitive | Less sensitive |
| 3BNC117 bNAb  VRC01 bNAb  VRC03 bNAb | gp120 CD4-binding site | Sensitive | Less sensitive | nd |
| F105 MAb | gp120 CD4-binding site | Resistant | Sensitive | nd |
| PGT151 | gp120-gp41 hybrid epitope | Less sensitive | More sensitive | nd |
| 4E10 | gp41 epitope | Less sensitive | More sensitive | nd |
| T20 | gp41 HR1 coiled coil | Resistant | Resistant | Sensitive |

nd - not determined.
